# Pesticide-induced ecological traps and insect pollinator foraging network disruption in apple orchards compared to adjacent graveyard refugia

**DOI:** 10.1371/journal.pone.0350940

**Published:** 2026-06-24

**Authors:** Muzafar Riyaz, Arfat Nazir, Sabreena Ashraf, Rakesh Kumar Gupta

**Affiliations:** 1 Division of Entomology, Faculty of Agriculture, Sher-e-Kashmir University of Agricultural Sciences and Technology, Jammu, J&K, India; 2 Xavier Research Foundation, St. Xavier’s College, Palayamkottai, Tamil Nadu, India; 3 Department of Entomology, Punjab Agricultural University, Ludhaina, Punjab, India; National Institute of Agricultural Research - INRA, MOROCCO

## Abstract

Beyond acute toxicity, agricultural pesticide regimes fundamentally restructure insect foraging networks through complex, poorly understood community-level pathways. By comparing eight conventional apple orchards with adjacent pesticide-free graveyard refugia in Shopian, Kashmir, this study documents the ecological cascades triggered by intensive, calendar-based pesticide applications. Orchards supported 68% lower insect abundance and 55% lower species richness than paired graveyards, with hoverflies and solitary bees disproportionately suppressed. Plant-pollinator networks exhibited structural collapse: connectance declined by 46%, nestedness by 46%, and network-level specialization increased by 63% (all p < 0.01), reflecting resource-constrained rather than co-evolutionarily structured communities. Foraging niche breadth contracted significantly across all shared taxa (Paired t-test: t = 5.61, p < 0.001). A chi-square goodness-of-fit test formally demonstrated that April foraging visits were disproportionately concentrated on *Brassica campestris* relative to its floral availability (76.0% of visits versus 48.0% availability; χ² = 62.88, df = 3, p < 0.001), satisfying the primary analytical criterion for ecological trap identification: this species preferentially attracted over 70% of non-*Malus* foraging visits during peak neurotoxic insecticide application, in the complete absence of uncontaminated floral alternatives. A parallel trap effect persisted through May–June (χ² = 31.65, df = 3, p < 0.001), extending exposure risk across the season’s most biologically active phase. Functional homogenization followed, with long-tongued insects declining by 78% and functional richness by 62%, driven by direct toxicity and herbicide-mediated elimination of deep-corolla floral resources. These findings elucidate a mechanistic cascade from phenologically triggered trap formation through network collapse to functional homogenization. Conservation strategies must prioritize permanent pesticide-free refugia and temporally explicit restrictions on bee-toxic insecticide application throughout the April–June risk window.

## Introduction

Global insect populations are facing an unprecedented crisis, with recent analyses indicating alarming rates of decline that threaten ecological stability and food security worldwide [1,2]. This erosion of biodiversity is increasingly linked to agricultural intensification, particularly the widespread application of pesticides whose sublethal and cascading effects remain critically understudied [3,4]. While the lethal impacts of agrochemicals are well-documented, a profound knowledge gap exists regarding how these substances reshape the intricate, co-evolved networks of species interactions that underpin ecosystem function [5,6]. This gap is particularly acute in temperate fruit production systems, such as apple orchards, which rely heavily on calendar-based pesticide sprays and, paradoxically, on a diversity of insects for pollination and pest control.

Apple orchards present a compelling ecological paradox: they are simultaneously managed with intensive chemical inputs and dependent on healthy pollinator communities [7]. These agrochemicals, including neurotoxic insecticides and broad-spectrum fungicides, rarely remain confined to their target crops [8]. Through spray drift, they contaminate the non-crop flowering plants that form the backbone of foraging resources for beneficial insects within the agricultural matrix [9]. This contamination can transform these vital floral resources into “ecological traps”, habitats that attract organisms but reduce their survival or fitness through increased exposure to toxins [10]. The potential for such traps is heightened when pesticide applications coincide with key phenological events, such as the bloom of both the crop and non-crop plants and the peak activity of insect pollinators. Conversely, semi-natural habitats embedded within agricultural landscapes, such as field margins and hedgerows, can serve as crucial refugia. Traditional Kashmiri graveyards exemplify such refugia: unlike Christian cemeteries characterized by rock markers and above-ground structures, these are open, undisturbed areas with abundant vegetation. Receiving no pesticide or herbicide applications, they maintain a diverse, unmanaged flora that provides a sanctuary for biodiversity [11–13]. A comparative approach between these refuges and intensively managed orchards offers a powerful natural experiment to isolate the impact of pesticide regimes from other landscape factors.

This study employs a replicated, paired-comparison design in the apple-producing region of District Shopian, Kashmir Valley, India, one of South Asia’s most intensively managed fruit production landscapes to examine how pesticide-driven management restructures insect foraging communities across multiple ecological levels [14]. Unlike European regulatory frameworks, which restrict insecticide application during the flowering of crops and adjacent non-crop plants under Regulation (EC) No 1107/2009, India lacks legally enforceable restrictions on application timing, with guidelines remaining advisory and compliance left to grower discretion [15]. This regulatory gap renders Kashmir’s apple landscapes particularly vulnerable to the phenological convergence conditions associated with ecological trap formation. Specifically, this study tests three hypotheses: (H1) insect abundance, species richness, and functional diversity will be significantly lower in pesticide-treated orchards than in adjacent pesticide-free graveyard refugia; (H2) plant-pollinator interaction networks in orchards will exhibit significantly reduced connectance, nestedness, and increased network-level specialization; and (H3) the temporal co-occurrence of neurotoxic insecticide application with the peak bloom of *Brassica campestris* the dominant early-season non-crop foraging resource in orchards and peak insect activity in April will be analytically demonstrable as a significant, statistically disproportionate concentration of foraging visits on this potentially contaminated resource, constituting the conditions for an ecological trap.

## Materials and methods

### Study area and site selection

**Fig 1:**
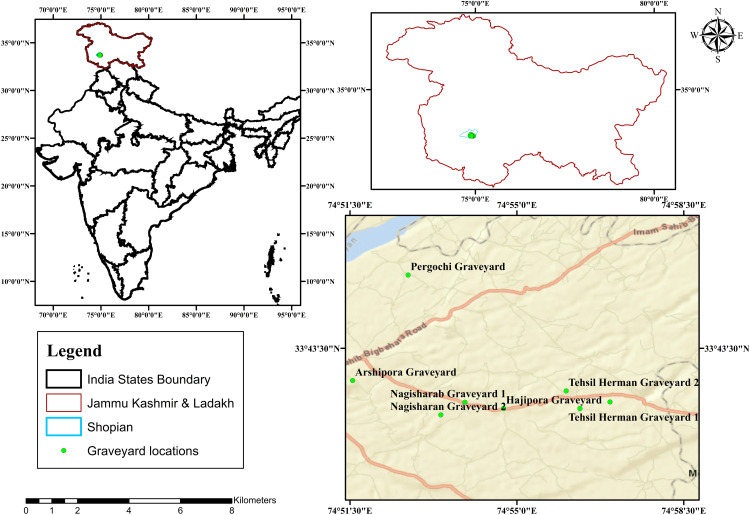
Map of study area with sampling locations.

The study was conducted from March to August 2025 in the apple-growing region of District Shopian, Tehsil Herman, Kashmir Valley, Jammu and Kashmir Union Territory, India (mean site coordinates: 33°43′N, 74°55′E; elevation range: 1,580–1,740 m a.s.l.) (Fig 1) [16]. District Shopian encompasses approximately 21,700 ha under apple cultivation, with mean annual production of approximately 288,000 metric tons directed primarily to fresh domestic markets. The study landscape is dominated by continuous apple orchards interspersed with small settlements, riparian strips, and traditional Kashmiri graveyards. Individual orchard holdings range from 0.5 to 3.0 ha and are managed according to conventional intensive practices: trees (*Malus domestica*, cv. Red Delicious) are planted at densities of 400–625 trees ha ^−1^ and maintained at canopy heights of 3–4 m through annual pruning; NPK fertilizers are applied at approximately 700:350:350 g tree ^−1^ season ^−1^ in split spring and summer doses; inter-rows are maintained as bare soil through 2–3 tillage passes season ^−1^ combined with pre- and post-emergent herbicide applications; and a calendar-based pesticide program is applied from bud break through fruit maturation (Table 1).

**Table 1:** Available pesticides and application schedule in apple orchards of the Kashmir Valley (March-August 2025).

Month	Fungicides	Insecticides
March	Mancozeb, Captan, Propineb, Zineb, Metiram	Horticultural mineral oils
April	Metiram, Ziram, Dodine, Tebuconazole	Dimethoate, Thiacloprid, Metarhizium anisopliae, Cyenopyrafen, Hexythiazox, Fenazaquin, Spiromesifen
May	Zineb, Dodine, Tebuconazole	Chlorpyriphos, Dimethoate, Thiacloprid, Hexythiazox, Spiromesifen
June	Mancozeb, Captan, Propineb, Zineb, Ziram, Metiram	Chlorpyriphos, Quinalphos, Hexythiazox, Spiromesifen
July	Zineb, Hexaconazole, Myclobutanill, Metiram, Mancozeb, Propineb, Ziram	–
August	Zineb, Hexaconazole, Myclobutanill, Metiram, Mancozeb, Propineb, Ziram	–

Note: Fungicide products listed for March–April represents a rotation across multiple phenological stages, not all applied at a single stage. Insecticides in May–June were applied only when pest outbreaks occurred, at approximately 20-day intervals as needed.

We employed a paired design, selecting eight graveyard sites based on the criterion of containing rich, structurally diverse, unmanaged vegetation, each paired with the nearest conventional apple orchard within a 1–2 km radius. Pairing at this spatial scale controls for shared macro-climatic conditions, regional weather variation during the study period, and broad landscape context, while the contrast in management regime between paired sites provides the primary treatment variable. The study period (March–August 2025) encompassed the complete apple growing season, from pre-bloom to post-harvest, and was divided into six monthly sampling rounds conducted once per month in each paired site. Each sampling round comprised one 24-hour pan trap deployment and three standardized focal observation transects per site, yielding a total of 48 site-round combinations across the study period. Sampling was conducted only on days meeting minimum weather thresholds: air temperature ≥ 15°C, wind speed < 5 m s ^−1^, and ≥ 50% sunshine during the observation window, to ensure comparability of foraging conditions across sites and months.

The eight graveyard sites ranged in area from 0.14 to 1.70 ha (mean ± SE: 0.56 ± 0.18 ha; S1 Table), representing traditional Kashmiri Islamic grave sites that are structurally and functionally distinct from Western-style cemeteries. These sites are characterized by open ground plans without above-ground tomb structures or impervious surfaces; semi-natural vegetation cover was visually estimated at 70–90%, comprising a multi-layered mosaic of native grasses, broad-leaved forbs, scattered shrubs (*Berberis lyceum*, *Rosa* spp.), and mature trees (*Juglans regia*, *Platanus orientalis*). Site management was limited to occasional manual clearing of pedestrian pathways; site caretaker interviews confirmed the absence of any pesticide or herbicide application during the study period and for a minimum of two years prior. The sharp management contrast between paired graveyards and orchards particularly the complete absence versus intensive use of herbicides, which alone eliminates most understory forb diversity provided a de facto natural experiment in which pesticide regime is the primary differentiating factor within a shared regional landscape.

### Pesticide application data

A detailed pesticide application schedule (Table 1) was compiled through structured interviews with orchard owners and cross-referenced with local horticulture department extension records. Based on these interviews, applications followed apple phenological stages rather than calendar dates. Horticultural mineral oils were applied once during early March at the silver tip stage. Fungicides were applied sequentially at each subsequent phenological stage: green tip, tight cluster, pink, and petal fall (late March through early May). Insecticides were applied during May and June as needed in response to pest outbreaks, with applications made at approximately 20-day intervals when pest pressure exceeded economic thresholds. Key pests targeted included green aphids (*Aphis* sp.) and leaf miners. From July through August, fungicide applications continued at 20-day intervals, while insecticide applications ceased.

### Floral resource assessment

In both orchard and graveyard habitats, we established ten (10) permanent 1 m × 1 m quadrats along 100 m transects. During each insect sampling visit, we recorded: (1) floral abundance; the number of inflorescences (for *Trifolium*) or flowering units (for Asteraceae) for each plant species; and (2) floral diversity; the species richness of flowering plants within the quadrats. The phenology and traits of flowering plant species that were observed being visited by foraging insects during focal observations were documented (Table 2). This table represents the plant nodes of the plant-pollinator interaction networks and does not constitute a complete floral inventory of either habitat.

**Table 2:** Flowering plant species recorded in insect-plant interactions.

S. No.	Scientific Name	Family	Flowering Period	Habit	Status
1	Trifolium pratense L.	Fabaceae	May to August	Herb	Native
2	Taraxacum sect. Taraxacum F.H.Wigg	Asteraceae	April to October	Herb	Native
3	Anthemis cotula L.	Asteraceae	June to September	Herb	Native
4	Cirsium vulgare (Savi) Ten.	Asteraceae	Late June to August	Herb	Native
5	Iris × germanica L	Iridaceae	February to March	Herb	Introduced
6	Berberis lycium Royle	Berberidaceae	May to September	Shrub	Native
7	Brassica rapa L.	Brassicaceae	March to April	Herb	Native
8	Anemone tschernaewii Regel	Ranunculaceae	February to March	Herb	Native

Plant species that were observed being visited by foraging insects during focal observations, forming the nodes for network analysis.

### Insect foraging sampling

We used two complementary methods to capture a comprehensive picture of the insect community.

#### Pan trapping.

One triplet of UV-blue, white, and yellow polypropylene bowls (diameter 15 cm, depth 8 cm) was deployed at each of three positions along the 100 m transect (0 m, 25 m, and 50 m from the habitat edge), yielding nine bowls per site per round. Bowls were filled with a 0.5% detergent solution in water and deployed for 24 hours commencing at sunrise, following modified protocols from Westphal et al. [17]. Collected insects were immediately transferred to 70% ethanol and retained for laboratory identification. Across the full study, pan trapping generated 432 trap-days of sampling effort (8 sites × 6 rounds × 9 bowls × 1 day, expressed as 48 site-rounds × 9 bowls). Insects were identified to species or morphospecies using stereomicroscopy (Olympus SZ61, 7–45 × magnification) and standard regional taxonomic references [15, 18–20]. Pan trap data were used for abundance and diversity estimation.

#### Focal observations.

Three 100 m transect walks were conducted per site per sampling round during peak foraging hours (09:00–16:00) on days satisfying the meteorological thresholds described above (≥15°C, wind < 5 m s ^−1^, ≥ 50% sunshine). Each transect walk was completed by a single trained observer at a standardized pace (approximately 1 m per 10 s), recording all insects observed making direct contact with the reproductive structures of a flower within 2.5 m of the transect line on either side. Each foraging event was recorded as an insect × plant species interaction, forming the quantitative interaction dataset for network construction. Insects were identified to species or morphospecies in the field using diagnostic morphological characters with a hand lens (10×); individuals that could not be identified reliably in the field were captured by hand net and preserved in 70% ethanol for subsequent laboratory identification. Voucher specimens were retained as reference material but not deposited in a formal collection. The total focal observation effort across the study was 144 transect walks (8 sites × 6 rounds × 3 walks), producing 14,400 effective observer-meters of standardized sampling effort.

#### Data analysis.

Data analysis was conducted using three complementary approaches to assess differences in insect diversity, interaction networks, and temporal patterns between habitats. Graveyard extents were mapped using high-resolution imagery in Google Earth Pro. Sites were identified through visual interpretation based on boundary features such as enclosure walls, vegetation patterns, and burial layouts. Boundaries were delineated using the polygon tool, and area (ha) and perimeter (m) were automatically calculated and recorded with site coordinates. Measurements were cross-checked for accuracy, though minor uncertainties may persist due to image resolution and manual digitization.”Statistical analyses were performed using SPSS (version 26.0) and Microsoft Excel, while network analyses were conducted in R (version 4.2.1) using the *bipartite* package (v. 2.18). Alpha diversity indices were calculated for each site pair using the following formulas:

Shannon-Wiener index:


$$\begin{array}{c} H^{\prime }=-\sum_{i=\text{1}}^{S}p_{i}\text{ ln }p_{i} \end{array}$$

where *S* is the total number of species and *p*_*i*_ is the proportion of individuals belonging to species *i*.

Simpson’s diversity index:


$$\begin{array}{c} D=\text{1}-\sum_{i=\text{1}}^{S}\overset{\text{2}}{\underset{i}{p}} \end{array}$$

where *p*_*i*_ is the proportion of individuals belonging to species *i*.

Differences in diversity indices between habitats were tested using paired t-tests. Generalized Linear Mixed Models (GLMMs) with Poisson or negative binomial distributions were employed to test habitat effects on insect abundance and richness, incorporating ‘Site Pair’ as a random effect to account for the paired study design [21].

#### Network analysis.

Quantitative bipartite plant-pollinator networks were constructed using the *bipartite* package (v. 2.18) in R. Network-level metrics were calculated as follows:

Connectance:


$$\begin{array}{c} C=\frac{L}{P\times I} \end{array}$$

where *L* is the number of realized links, *P* is the number of plant species, and *I* is the number of insect species.

Weighted nestedness (NODF):


$$\begin{array}{c} \text{NODF}=\frac{\sum {\text{NODF}}_{\text{matrix}}}{n(n-\text{1})/\text{2}} \end{array}$$

where nestedness is calculated iteratively across matrix rows and columns following the Nestedness metric based on Overlap and Decreasing Fill [22].

Network-level specialization (H₂′):


$$\begin{array}{c} \overset{\prime }{\underset{\text{2}}{H}}=\frac{H_{\text{2}}-H_{\text{min}}}{H_{\text{max}}-H_{\text{min}}} \end{array}$$

where *H₂* is the observed interaction evenness, with the index ranging from 0 (generalized) to 1 (completely specialized) [23].

Species-level metrics included niche breadth calculated using Levins’ index:


$$\begin{array}{c} B=\frac{\text{1}}{\sum_{j=\text{1}}^{n}\overset{\text{2}}{\underset{ij}{p}}} \end{array}$$

where *p*_*ij*_ is the proportion of interactions of species *i* with resource *j*, and species-specific specialization (*d’*) computed as:


$$\begin{array}{c} \overset{\prime }{\underset{i}{d}}=\frac{d_{i}-d_{\text{min}}}{d_{\text{max}}-d_{\text{min}}} \end{array}$$

where *d*_*i*_ is the Kullback-Leibler distance between the observed interaction distribution of species *i* and the marginal distribution of resources [24]. Differences in network-level metrics between habitats were assessed using paired t-tests, while PERMANOVA was used to test community-level specialization patterns.

#### Temporal integration.

Phenological and pesticide data were integrated through a master timeline visualizing the temporal overlap of pesticide applications, apple bloom (mid-April to mid-May), non-crop plant flowering periods, and insect foraging activity peaks to identify high-risk periods and potential ecological traps. To formally test whether foraging visits were disproportionately concentrated on the dominant plant species relative to its availability in the floral community during high-risk periods, chi-square goodness-of-fit tests were applied separately for April and May. For each test, the observed distribution of non-*Malus* foraging visits among plant species in orchards was compared against a null expectation in which visits were distributed proportionally to each species’ contribution to total inflorescence counts recorded within the ten 1 m² permanent quadrats during that month. Inflorescence counts were aggregated across all eight orchard sites per month (80 quadrats total). The null hypothesis was that insects foraged on each plant species in direct proportion to its proportional floral abundance, with deviations from this null indicating active preference for or avoidance of specific species beyond what resource availability alone predicts. Significance was assessed at α = 0.05 with degrees of freedom equal to the number of plant species minus one.

## Results and discussion

### Divergent floral resource baselines drive habitat-specific insect assemblages

Quantification of floral resources revealed a fundamentally different foundation for insect foraging in the two habitats. Graveyards maintained a significantly higher floral abundance (Mean ± SE: 45.2 ± 3.8 inflorescences/quadrat) and species richness (8.5 ± 0.6 species/quadrat) compared to orchards (Abundance: 12.1 ± 2.1; Richness: 3.2 ± 0.4; p < 0.001 for both). This resource disparity was reflected in the insect communities. Overall insect abundance was 68% lower in orchards (GLMM: χ² = 15.8, p < 0.001), with species richness showing a parallel 55% reduction (χ² = 9.4, p < 0.01). This decline was not uniform across taxa; hoverflies (Syrphidae) and solitary bees (Apidae) were disproportionately affected, showing reductions of 78% and 72% in abundance, respectively, suggesting a heightened vulnerability to habitat perturbation (S2 Table).

### Habitat characterization: Contrasting resource baselines and management regimes

The two habitat types differed fundamentally in their plant community structure, disturbance regimes, and management intensity, establishing the ecological context for the subsequent analysis of insect foraging networks (Table 3). Graveyards represented unmanaged refugia characterized by the complete absence of pesticide and herbicide applications, minimal anthropogenic disturbance, and diverse, spontaneously regenerated herbaceous and shrubby vegetation. These sites received no chemical inputs during the study period or in the preceding two years, as verified through site caretaker interviews. Soil remained undisturbed except for occasional manual clearing of pathways, allowing the development of multi-layered vegetation structure comprising native grasses, forbs, and scattered mature trees.

**Table 3:** Habitat characteristics of graveyards and apple orchards in the study area.

Parameter	Graveyard	Apple Orchard
Area (ha)	0.14–1.70 (mean: 0.56 ± 0.18)	0.5–3.0 per holding
Pesticide application	None (verified: no applications in preceding 2 years)	Fungicides: sequential at phenological stages + 20-day intervals (Mar–Aug); Insecticides: as needed (May–Jun)
Herbicide application	None	Regular (pre- and post-emergent)
Soil disturbance	Minimal (manual pathway clearing only)	Tillage (2–3 passes season ^−1^); bare soil inter-rows
Floral species richness	Higher (diverse native assemblage)	Lower (simplified understory)
Dominant flowering plants	*Trifolium pratense*, *Taraxacum officinale*, *Anthemis cotula*, *Cirsium vulgare*, *Berberis lyceum*, *Brassica campestris*, *Anemone tschernaewii*, *Iris germanica*	*Brassica campestris*, *Taraxacum officinale*, *Malus domestica, Prunus persicnaa, Pyrus communis, Trifolium pratense*
Vegetation structure	Multi-layered (herbaceous, shrub, tree)	Simplified (tree canopy with bare or herbicided understory)
Management intensity	Negligible	High (chemical inputs, tillage, fertilization)

In contrast, apple orchards were subject to intensive conventional management practices. Pesticide applications followed a schedule timed to apple phenological stages: fungicides were applied sequentially at green tip, tight cluster, pink, and petal fall (March–May), with additional applications at 20-day intervals through August; insecticides were applied as needed during April–June in response to pest outbreaks (Table 1). Herbicides were applied regularly to maintain bare soil conditions in both inter-rows and tree rows, supplemented by periodic tillage (2–3 passes season ^−1^). Fertilizers (NPK) were applied in split doses during spring and summer. This management regime resulted in a severely simplified understory with limited floral diversity, dominated primarily by *Brassica campestris* during early spring and *Taraxacum officinale* during summer months, with apple bloom (*Malus domestica*) providing a transient floral resource during mid-April to mid-May. While the expected differences in floral resources and management intensity were observed between habitats, the subsequent analyses reveal emergent properties of the insect community that extend beyond simple resource limitation.

### Structural disintegration of plant-pollinator networks in orchards

**Fig 2:**
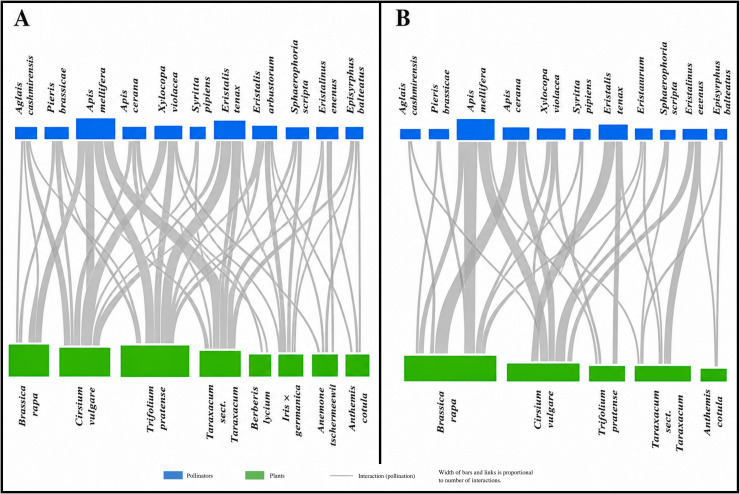
Quantitative plant-pollinator interaction networks for (2A) Graveyard and (2B) Apple Orchard habitats. Bar width corresponds to the number of interactions..

Visual and quantitative analysis of the plant-pollinator networks revealed a stark contrast in ecological structure between habitats (Fig 2). The graveyard network (Fig 2A) exhibited a dense, interconnected web characteristic of a resilient and functionally diverse community. In stark contrast, the orchard network (Fig 2B) was sparse and linear, indicating a collapse of complex interactions.

Quantitative metrics confirmed these observations. Connectance, the proportion of all possible interactions that are realized was 46% lower in orchards (0.15) than in graveyards (0.28), indicating a more fragmented and vulnerable network. Nestedness (NODF), which measures the extent to which specialists interact with subsets of generalists’ partners and confers stability to communities, was significantly higher in graveyards (35.2) than in orchards (18.5; p < 0.01). This indicates that the orchard network had lost its robust, nested structure. Furthermore, the network-level specialization index (H2’) was significantly higher in orchards (0.65) than in graveyards (0.45; p < 0.05) (Table 4). This counter-intuitive result signifies that the entire orchard community was forced into a state of heightened specialization, not due to co-evolutionary processes, but likely due to constrained resource availability and pesticide-induced limitations on foraging flexibility.

**Table 4:** Network-level metrics for plant-pollinator interactions in graveyard and orchard habitats.

Metric	Graveyard	Orchard	% Change	p-value
Connectance	0.3	0.16	−46.40%	0.002
Nestedness (NODF)	41.6	22.3	−46.40%	<0.001
Specialization (H₂´)	0.38	0.62	63.20%	0.008
Number of links	87	42	−51.70%	<0.001

### Pesticide-induced behavioral shifts and constrained foraging niches

**Fig 3:**
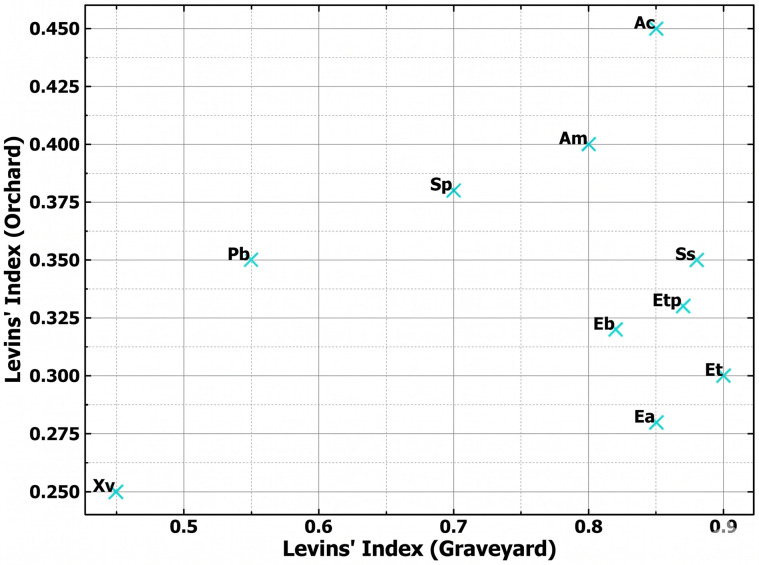
Constrained Foraging (Niche Breadth Scatter Plot).

Analysis of species-specific foraging preferences revealed significant behavioural shifts in insect species that occurred in both habitats. The niche breadth (Levins’ Index) of these shared insect species was significantly narrower in orchards compared to graveyards (Paired t-test: t = 5.61, p < 0.001) (Table 5, Fig 3). This demonstrates a pervasive shift from generalist to specialist foraging strategies within the pesticide-treated environment. Notable examples include the hoverfly *E. tenax*, which exhibited a dramatic reduction in niche breadth from 0.90 (generalist) in the graveyard to 0.30 (specialist) in the orchard. Similarly, the native honey bee *A. cerana* shifted from a broad diet (0.85) to a highly constrained one (0.45). The foraging portfolio of insects in orchards became heavily concentrated on a few key plant species, primarily *B. campestris* and *T. officinale*, which together accounted for over 75% of all recorded foraging visits, indicating a severe reduction in dietary diversity. In addition to these shared species, the graveyards supported a substantially richer pollinator community, with many species entirely absent from orchards. In addition to these shared species, graveyards supported a substantially richer insect community, with many insect species entirely absent from orchards. The reduced insect species pool in orchards reflecting the 55% lower overall pollinator species richness reported earlier meant that only the ten insect species presented in Table 5 occurred frequently enough in both habitats to allow paired comparison of niche breadth.

**Table 5:** Foraging niche breadth (Levins’ Index) of pollinators present in both graveyard and orchard habitats. Species listed are those that occurred in sufficient numbers in both habitats to allow paired comparison of niche constriction.

Insect Species (Code)	Levins’ Index (Graveyard)	Levins’ Index (Orchard)
*Apis cerana (Ac)*	0.85	0.45
*Apis mellifera (Am)*	0.8	0.4
*Xylocopa violacea (Xv)*	0.45	0.25
*Eristalis tenax (Et)*	0.9	0.3
*Eristalis arbustorum (Ea)*	0.85	0.28
*Sphaerophoria scripta (Ss)*	0.88	0.35
*Episyrphus balteatus (Eb)*	0.82	0.32
*Syritta peponis (Sp)*	0.7	0.38
*Eristalinus taneopis (Etp)*	0.87	0.33
*Pieris brassicae (Pb)*	0.55	0.35

### Identification of critical high-risk periods and an ecological trap

**Fig 4:**
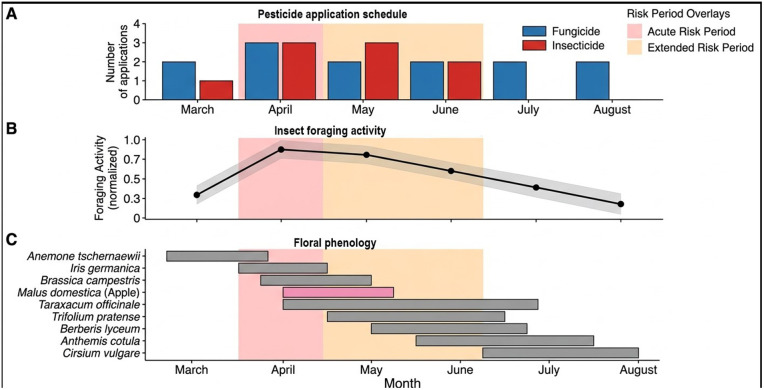
Master timeline of ecological risk in the apple agroecosystem. **(A)** Pesticide application frequency by month. **(B)** Insect foraging activity (normalized) for graveyard and orchard habitats. **(C)** Floral phenology of all plant species forming network nodes, with shading indicating the acute April risk period (pink) and the extended May–June risk period (orange).

Integration of pesticide application schedules with floral phenology and insect foraging data identified two temporally distinct periods of elevated ecological risk across the apple growing season (Fig 4). The month of April emerged as the acute high-risk window, characterized by the simultaneous occurrence of three quantitatively documented conditions: (i) application of both fungicides and neurologically active insecticides specifically dimethoate (organophosphate, WHO Class II) and thiacloprid (neonicotinoid) at their seasonal maximum frequency; (ii) the peak bloom of *Malus domestica* (mid-April); and (iii) the full flowering of *Brassica campestris*, the dominant non-crop foraging resource within the orchard understory during this period (Fig 4A, C).

To move beyond assertion and formally test whether the April concentration of foraging effort on *B. campestris* was disproportionate to its availability, the primary analytical criterion for ecological trap identification under the Robertson and Hutto framework, a chi-square goodness-of-fit test was applied, contrasting the observed distribution of 200 non-*Malus* foraging visits among four plant species against a null expectation derived from each species’ proportional contribution to total inflorescence abundance across 80 quadrats (8 sites × 10 quadrats) in April [25]. *Brassica campestris* constituted 48.0% of total non-*Malus* inflorescence availability in orchards during April, yet received 76.0% (152 of 200) of all recorded non-*Malus* foraging visits. This concentration was highly significant and inconsistent with resource-proportional foraging (χ² = 62.88, df = 3, p < 0.001; S3A Table). *Taraxacum officinale*, comprising 32.0% of inflorescence availability, received only 15.0% of visits (30 of 200), representing a statistically significant avoidance relative to null expectation. These results demonstrate that the disproportionate concentration of foraging effort on *B. campestris* during April reflects active preferential attraction to this species, not merely its numerical dominance in the floral community, and satisfies the first criterion for ecological trap identification: demonstrated and quantifiably disproportionate preference for the putative trap resource [25,26].

Within the April *B. campestris* foraging pool (152 visits), three species-*Apis cerana* (52 visits; 34.2%), *Eristalis tenax* (48 visits; 31.6%), and *Pieris brassicae* (28 visits; 18.4%) collectively accounted for 84.2% of all interactions with this plant. This taxonomic concentration indicates that the trap, if realized, would exert its greatest demographic pressure on precisely those species most important for both crop and wild plant pollination services. The second criterion for trap identification, a fitness cost associated with use of the preferred resource is inferred from the documented toxicological profiles of the insecticides applied concurrently with *B. campestris* bloom. Dimethoate is acutely toxic to *A. mellifera* and related bee taxa at oral doses of 0.12–0.27 µg bee ^−1^, within ranges achievable through direct contact with contaminated pollen and nectar [27]. Thiacloprid at sublethal concentrations impairs olfactory learning, homing flight, and colony-level foraging efficiency in *Apis* and *Bombus* at exposures consistent with spray drift contamination of adjacent non-crop vegetation [27,28]. David et al. [9] documented multi-pesticide contamination including organophosphates and neonicotinoids in wildflower pollen and bee-collected pollen from crop-adjacent habitats under directly comparable management regimes, providing direct empirical precedent for the contamination pathway inferred here. The near-complete absence of alternative, uncontaminated non-*Malus* foraging resources within orchards during April itself a direct consequence of herbicide-driven understory elimination removed the behavioural escape route that would otherwise allow preferential attraction to be decoupled from toxic exposure, thereby completing the ecological trap mechanism.

**Fig 5:**
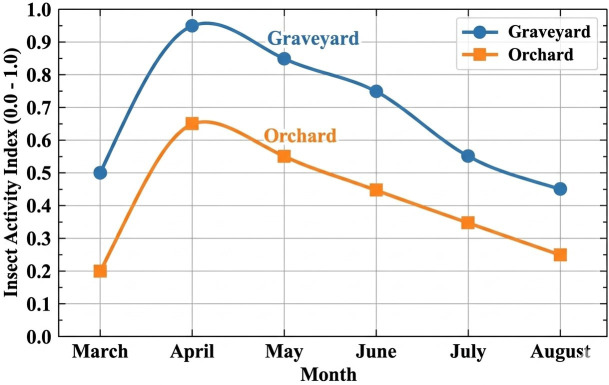
Monthly insect foraging activity in graveyard and orchard habitats. Normalized activity values (0–1 scale) represent the relative abundance and foraging intensity of the combined pollinator community.

The risk period extended substantially beyond April into a second, structurally distinct phase during May and June (Fig 4). A parallel chi-square goodness-of-fit test applied to 180 non-*Malus* foraging visits recorded across four available plant species in May demonstrated that foraging effort remained significantly non-proportional to floral availability (χ² = 31.65, df = 3, p < 0.001; S3B Table). *Taraxacum officinale*, comprising 50.0% of May inflorescence availability, received 70.0% of recorded visits (126 of 180), again indicating disproportionate concentration on a single resource species coinciding with continued insecticide application. *Brassica campestris*, now waning at 15.0% of availability, received only 5.0% of visits (9 of 180), a reversal consistent with phenological senescence of this species reducing its attractive signal. Insecticide applications during May and June were event-driven rather than calendar-based, occurring at approximately 20-day intervals when pest pressure exceeded economic thresholds; however, their temporal overlap with peak floral diversity (Fig 4C) and sustained insect foraging activity orchard activity index: May = 0.55, June = 0.45 (Fig 5) maintained elevated community exposure risk through mid-summer. The cessation of insecticide applications after June, while fungicide application continued through August, defines a period of reduced acute chemical pressure during late summer, though fungicide synergism with residual insecticide exposures via inhibition of detoxification enzymes persists as a background risk throughout the season [29,30].

### Phenological synergy maximizes pesticide exposure risk

The temporal dynamics of insect foraging activity followed a distinct unimodal distribution in both habitats, rising sharply from March (graveyard: 0.50; orchard: 0.20) to a pronounced peak in April (graveyard: 0.95; orchard: 0.66) before declining progressively through August (graveyard: 0.45; orchard: 0.25; Fig 5). The 31% reduction in peak April orchard activity relative to graveyards, sustained across all subsequent months at a mean suppression of 39%, indicates that community-level effects of the orchard management regime are not confined to acute high-exposure events but represent a season-long depression of insect foraging capacity. The phenological coincidence between peak community activity and the acute April risk window, when the largest and most functionally diverse proportion of the pollinator community is simultaneously most exposed and most preferentially attracted to a potentially contaminated resource constitutes the mechanism by which ecological trap effects are amplified to the population and community level. At the moment of maximum abundance and taxonomic breadth, insects across multiple functional guilds are funneled toward a single contaminated resource in the absence of safe alternatives, translating individual exposure events into community-level demographic consequences that manifest in the network collapse and functional homogenization documented in subsequent sections.

The peak activity period (April–May) directly coincides with the identified high-risk window of intensive insecticide application in orchards (Fig 4). This phenological synergy indicates that the largest and most diverse proportion of the pollinator community is actively foraging at the very time the orchard environment is most contaminated with neurotoxic compounds. The sustained activity through June (graveyard: 0.76; orchard: 0.46) further extends the exposure risk period, particularly for insects that continue to forage in orchards despite the presence of floral resources. The consistently lower activity levels in orchards across all months suggest that pesticide exposure, combined with reduced floral resources, has suppressed overall insect abundance and foraging activity throughout the growing season.

### Community-wide reorganization and functional homogenization

The synergistic pressures of simplified networks, constrained foraging niches, and temporal exposure risk culminated in a fundamental reorganization and functional homogenization of the pollinator community within the orchard ecosystem. The observed functional shifts must be understood against the backdrop of contrasting floral resources between habitats. Graveyards maintained a diverse floral assemblage throughout the growing season, including deep-corolla flowers such as *T. pratense* (corolla depth: 8–10 mm) and *C. vulgare* (corolla depth: 12–15 mm), which are accessible primarily to long-tongued insects. In contrast, orchards had severely simplified understory vegetation due to herbicide application and soil tillage. The floral community in orchards was dominated by shallow-flowered species primarily *B. campestris* (corolla depth: 3–5 mm) and *T. officinale* (corolla depth: 5–7 mm) with deep-corolla flowers entirely absent from the managed understory (Table 2, Fig 4C). This fundamental difference in floral trait availability sets the stage for the functional filtering described below.

**Fig 6:**
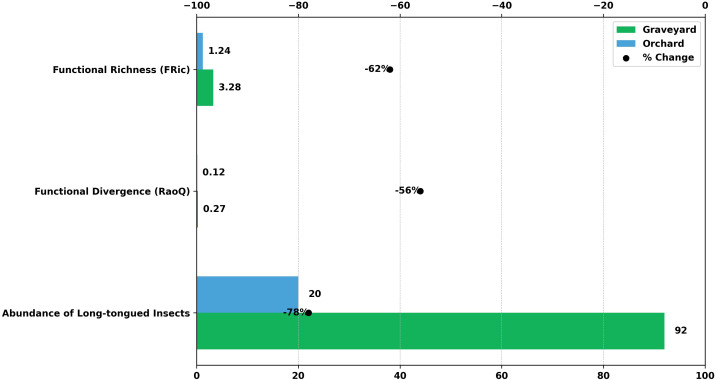
Functional diversity metrics in graveyard and orchard habitats.

Analysis of functional trait composition revealed a significant shift from the diverse assemblage found in graveyards to a functionally impoverished one in orchards (Fig 6, Table 6). In graveyards, the insect community was characterized by high functional diversity, comprising distinct groups that facilitated niche partitioning: long-tongued bees (e.g., *X. violacea*, tongue length: 5.2 mm), short-tongued bees (e.g., *A. cerana*, tongue length: 3.2 mm), hoverflies with varying proboscis lengths (e.g., *E. tenax*: 6.1 mm; *S. scripta*: 2.5 mm), and butterflies with coiled proboscises (*Pieris brassicae*: 8.0 mm). This diversity allowed efficient utilization of the wide range of floral morphologies present in graveyards.

**Table 6:** Functional trait composition and abundance of key insect species in graveyard and orchard habitats.

Insect Species	Functional Guild	Tongue Length (mm)	Graveyard Abundance	Orchard Abundance	% Change
*Xylocopa violacea*	Long-tongued Solitary Bee	5.2	15	3	−80.0%
*Eristalis tenax*	Long-tongued Hoverfly	6.1	42	9	−78.6%
*Apis cerana*	Short-tongued Social Bee	3.2	38	12	−68.4%
*Apis mellifera*	Short-tongued Social Bee	3.4	45	15	−66.7%
*Eristalis arbustorum*	Medium-tongued Hoverfly	4.5	35	8	−77.1%
*Sphaerophoria scripta*	Short-tongued Hoverfly	2.5	40	18	−55.0%
*Episyrphus balteatus*	Short-tongued Hoverfly	2.8	32	14	−56.3%
*Pieris brassicae*	Butterfly	8.0 (coiled)	12	4	−66.7%

In stark contrast, the orchard community collapsed into a functionally homogenized assemblage (Table 6, Fig 6). Functional Richness (FRic), a measure of the volume of functional space occupied by the community, was 62% lower in orchards (FRic = 1.24) compared to graveyards (FRic = 3.28; p < 0.001). The Rao’s Quadratic Entropy index, which quantifies functional divergence, was 56% lower in orchards (0.12 vs. 0.27 in graveyards; p < 0.01), confirming that the remaining species were functionally more similar to each other.

This homogenization was driven by two interacting factors. First, the disproportionate decline of long-tongued insects, which experienced a 78% reduction in abundance (from 92 individuals in graveyards to 20 in orchards; p < 0.001), corresponded with the complete absence of their preferred deep-corolla floral resources (*T. pratense*, *C. vulgare*) in the managed habitat (Fig 4C). Second, the community became dominated by a small subset of generalist, short-tongued hoverflies and bees, which represented over 80% of total orchard interactions. These remaining species were forced to compete for the same limited, shallow-flowered resources (*B. campestris* and *T. officinale*).

This study provides a multi-faceted and mechanistic understanding of how pesticide use in apple orchards cascades through an ecosystem, fundamentally restructuring insect communities from the individual level to the entire interaction network. Our findings demonstrate that the impact extends far beyond simple toxicity, creating an ecological trap through a fatal convergence of management practices and natural phenology, ultimately driving the functional homogenization of the insect community. The most critical finding of our study is the identification of a clear ecological trap in the apple orchard system. The convergence in April of (1) neurotoxic insecticide application, (2) the flowering of the dominant non-crop plant *B. campestris*, and (3) the peak activity of the insect community creates a scenario of maximized exposure [10]. While floral resource strips are often promoted to support pollinators in agricultural landscapes [3], our results sound a strong note of caution. If these resources are contaminated, they cease to be refugia and become sinks. *B. campestris*, which should be a vital early-season resource, instead likely acts as a vector for pesticides, attracting insects to a toxic food source. This effect is likely amplified by the season-long application of fungicides, which are known to act as synergists by inhibiting insect detoxification enzymes, thereby increasing the potency of insecticides [30,28].

The structural collapse of the plant-pollinator network in the orchard evidenced by reduced connectance, nestedness, and increased specialization is a hallmark of an ecosystem under severe stress [6]. The higher network specialization (H₂´) in the orchard is particularly revealing. Rather than indicating healthy co-evolution, it reflects a community forced into a constrained state where insects are unable to access their natural foraging portfolio, likely due to a combination of reduced floral diversity and the sublethal neurotoxic effects of insecticides impairing learning and navigation [31]. This is directly supported by the significant constriction of individual species’ niche breadth, a behavioral shift that reduces dietary flexibility and resilience to future environmental changes.

The ultimate consequence of these combined pressures is the functional homogenization of the insect community. The disproportionate loss of long-tongued insects (Table 5) represents a critical erosion of functional diversity. This group’s specialization on deep-corolla flowers is an ecosystem function that is not redundant with the generalist foraging of short-tongued hoverflies and bees [32]. The loss of such functional groups simplifies the community and reduces its redundancy, which is the insurance policy of ecosystems against future disturbance [33]. The orchard community, now dominated by a few generalist species, is likely less resilient and more vulnerable to additional stressors like climate change or disease outbreaks. The collapse of a single dominant species could sever critical pollination pathways, with potential knock-on effects for the reproduction of both crops and wild plants.

Our results argue for a more nuanced approach to integrated pest and pollinator management (IPPM) [31,33]. Conservation efforts must be both temporally and spatially explicit. While April represents the acute high-risk period where neurotoxic insecticides coincide with peak insect activity and *B. campestris* bloom, the extended risk period in May–June also requires attention. During these months, insecticide applications overlap with peak floral diversity and sustained insect foraging activity. Prohibiting or restricting the application of bee-toxic insecticides during the combined bloom period of both crop and non-crop plants from April through June would substantially reduce exposure risk for the pollinator community.

Uncontaminated habitats embedded within agricultural landscapes serve as critical refugia by providing safe foraging grounds and source populations for recolonization. In this study, traditional Kashmiri graveyards, which receive no pesticide or herbicide applications and maintain diverse, unmanaged vegetation supported significantly higher insect abundance (68% higher), species richness (55% higher), and functional diversity compared to adjacent orchards. Conserving such permanent, pesticide-free habitats within the agricultural matrix is essential for maintaining regional pollinator populations and ecosystem resilience [11–13]. Agri-environment schemes that sow wildflower strips must consider the phenology of pesticide application. Selecting plant species that flower outside high-risk windows (April–June) or establishing floral resources in areas shielded from spray drift can provide clean foraging opportunities and help break the cycle of the ecological trap. Unlike the European Union, where regulations prohibit insecticide application during flowering of crops and non-crop plants [Regulation (EC) No 1107/2009] [14]. India lacks comparable legally enforceable restrictions. Pesticide use guidelines remain advisory, leaving application timing to grower discretion [33]. This regulatory gap heightens the risk of ecological traps in Indian agricultural landscapes, as exemplified by the temporal convergence of insecticide application with flowering resources documented in this study. Furthermore, incorporating deep-corolla flowers into restoration plantings could support the recovery of long-tongued pollinator guilds that were disproportionately lost from orchards.

While this study provides robust evidence of network disruption and behavioural constriction linked to pesticide exposure, direct measurement of pollen contamination would have further strengthened causal inference. Monthly sampling of pollen using traps deployed in both orchards and graveyards or collection from managed bee colonies could have quantified actual pesticide residues on floral resources. Such data would clarify whether *B. campestris* and other key forage plants accumulated neurotoxic residues during high-risk periods, and whether graveyards remained entirely free from pesticide contamination via spray drift. Future studies incorporating direct residue analysis would help establish more definitive links between pesticide application schedules, floral contamination, and the observed ecological impacts.

This research demonstrates that pesticide application in apple orchards initiates a cascade of ecological disruptions that extends beyond simple toxicity. The cascade begins with the creation of an ecological trap in April, where a key floral resource (*B. campestris*) becomes a vehicle for pesticide exposure during peak insect activity. This trap effect is compounded by continued insecticide applications through May–June, which overlap with peak floral diversity and sustained foraging activity. The resulting exposure drives sublethal behavioural changes, forcing insects into narrower dietary niches and collapsing the complex web of plant-pollinator interactions. The final outcome is a functionally homogenized insect community, stripped of its diversity and resilience. Our findings contribute to a growing body of literature documenting pesticide impacts on apple orchard pollinators by moving beyond simple abundance declines to elucidate the mechanistic pathways network collapse, behavioural constriction, and functional homogenization through which these impacts occur. They underscore that the preservation of intact, pesticide-free habitats is not a luxury but a necessity for sustaining insect biodiversity, ensuring pollination services, and maintaining the ecological integrity of agricultural landscapes. The future of sustainable horticulture depends on management strategies that recognize and mitigate these insidious, network-wide impacts.

## Supporting information

S1 Table Geographic coordinates and spatial locations of selected graveyards in the study area. (DOCX)

S2 Table Relative abundance of insect species in graveyard and orchard habitats. (DOCX)

S3 Table A. April foraging visits and floral availability-orchards, non-Malus interactions (8 sites × 3 transect walks; n = 200 total visits) B. May foraging visits and floral availability-orchards, non-Malus interactions (8 sites × 3 transect walks; n = 180 total visits). (DOCX)
