# Supplementary material for: Pesticide-induced ecological traps and insect pollinator foraging network disruption in apple orchards compared to adjacent graveyard refugia
Source: PLoS One. 2026 Jun 24;21(6):e0350940. doi: 10.1371/journal.pone.0350940 (PMC13293464; doi:10.1371/journal.pone.0350940)
Supplement: S1 Table — (DOCX) [file pone.0350940.s001.docx]

**S1 Table.** Geographic coordinates and spatial locations of selected graveyards in the study area.

| S. No. | Graveyard Name | Coordinates (DMS) | Area (ha) |
| --- | --- | --- | --- |
| 1 | Alamgunj Graveyard | 33°42′38.01″N 74°57′26.45″E | 1.70 |
| 2 | Tehsil Herman Graveyard 1 | 33°42′14.36″N 74°56′19.33″E | 0.42 |
| 3 | Tehsil Herman Graveyard 2 | 33°42′24.61″N 74°56′12.67″E | 0.38 |
| 4 | Hajipora Graveyard | 33°42′14.46″N 74°54′43.22″E | 0.14 |
| 5 | Nagisharan Graveyard 1 | 33°42′22.31″N 74°53′54.62″E | 0.15 |
| 6 | Nagisharan Graveyard 2 | 33°42′24.84″N 74°53′48.15″E | 0.27 |
| 7 | Arshipora Graveyard | 33°42′49.51″N 74°51′33.30″E | 0.97 |
| 8 | Pergochi Graveyard | 33°45′02.18″N 74°52′43.17″E | 0.28 |
