# Supplementary material for: Pesticide-induced ecological traps and insect pollinator foraging network disruption in apple orchards compared to adjacent graveyard refugia
Source: PLoS One. 2026 Jun 24;21(6):e0350940. doi: 10.1371/journal.pone.0350940 (PMC13293464; doi:10.1371/journal.pone.0350940)
Supplement: S2 Table — (DOCX) [file pone.0350940.s002.docx]

**S2 Table. Relative abundance of insect species in graveyard and orchard habitats.**

| Insect Species | Functional Guild | Graveyard Abundance | Orchard Abundance | % Change |
| --- | --- | --- | --- | --- |
| *Apis cerana* | Short-tongued social bee | 38 | 12 | –68.4% |
| *Apis mellifera* | Short-tongued social bee | 45 | 15 | –66.7% |
| *Xylocopa violacea* | Long-tongued solitary bee | 15 | 3 | –80.0% |
| *Eristalis tenax* | Long-tongued hoverfly | 42 | 9 | –78.6% |
| *Eristalis arbustorum* | Medium-tongued hoverfly | 35 | 8 | –77.1% |
| *Sphaerophoria scripta* | Short-tongued hoverfly | 40 | 18 | –55.0% |
| *Episyrphus balteatus* | Short-tongued hoverfly | 32 | 14 | –56.3% |
| *Syritta peponis* | Short-tongued hoverfly | 28 | 12 | –57.1% |
| *Eristalinus taneopis* | Medium-tongued hoverfly | 30 | 10 | –66.7% |
| *Pieris brassicae* | Butterfly | 12 | 4 | –66.7% |
| Other syrphids | Various hoverflies | 25 | 6 | –76.0% |
| Other solitary bees | Various bees | 18 | 3 | –83.3% |
| Other butterflies | Various butterflies | 10 | 2 | –80.0% |
| Total |  | 370 | 116 | –68.6% |

Values represent total individuals observed during focal observations and pan trapping across all sampling rounds (March–August). This table expands upon Table 5 by including additional taxa (Syritta peponis, Eristalinus taneopis, and aggregated groups) to provide a complete community inventory. Functional guild classification based on tongue/proboscis length and foraging behavior. % Change calculated as (Orchard-Graveyard)/Graveyard × 100.
