# Supplementary material for: Pesticide-induced ecological traps and insect pollinator foraging network disruption in apple orchards compared to adjacent graveyard refugia
Source: PLoS One. 2026 Jun 24;21(6):e0350940. doi: 10.1371/journal.pone.0350940 (PMC13293464; doi:10.1371/journal.pone.0350940)
Supplement: S3 Table — (DOCX) [file pone.0350940.s003.docx]

S3A Table. April foraging visits and floral availability-orchards, non-Malus interactions (8 sites × 3 transect walks; n = 200 total visits)

| Plant Species | Inflorescence units (80 quadrats) | Proportional availability | Observed visits | Expected visits | (O−E)²/E |
| --- | --- | --- | --- | --- | --- |
| *Brassica campestris* | 192 | 0.480 | 152 | 96.0 | 32.67 |
| *Taraxacum officinale* | 128 | 0.320 | 30 | 64.0 | 18.06 |
| *Anemone tschernaewii* | 52 | 0.130 | 11 | 26.0 | 8.65 |
| *Iris germanica* | 28 | 0.070 | 7 | 14.0 | 3.50 |
| Total | 400 | 1.000 | 200 | 200.0 | χ² = 62.88 |

df = 3; p < 0.001

S3B Table. May foraging visits and floral availability-orchards, non-Malus interactions (8 sites × 3 transect walks; n = 180 total visits)

| Plant Species | Inflorescence units (80 quadrats) | Proportional availability | Observed visits | Expected visits | (O−E)²/E |
| --- | --- | --- | --- | --- | --- |
| *Taraxacum officinale* | 120 | 0.500 | 126 | 90.0 | 14.40 |
| *Trifolium pratense* | 48 | 0.200 | 27 | 36.0 | 2.25 |
| *Berberis lyceum* | 36 | 0.150 | 18 | 27.0 | 3.00 |
| *Brassica campestris* | 36 | 0.150 | 9 | 27.0 | 12.00 |
| Total | 240 | 1.000 | 180 | 180.0 | χ² = 31.65 |

df = 3; p < 0.001
